# Identification of the barriers and enablers for receiving a speaking up message: a content analysis approach

**DOI:** 10.1186/s41077-023-00256-1

**Published:** 2023-07-06

**Authors:** Melanie Barlow, Kate J. Morse, Bernadette Watson, Fiona Maccallum

**Affiliations:** 1 Faculty of Health Sciences, Australian Catholic University, 1100 Nudgee Road, Banyo, QLD Australia; 2 School of Psychology, University of Queensland, St. Lucia, QLD Australia; 3 College of Nursing & Health Professions, Drexel University, 245 N 15Th Street, Mail Stop 501, 4Th Floor, Room 4606, Philadelphia, PA 19102 USA; 4 Department of English and Communication, Hong Kong Polytechnic University, Hung Hom, Kowloon, Hong Kong

**Keywords:** Speaking up, Receiver, Healthcare communication, Debrief, Simulation, Interprofessional education, Patient safety

## Abstract

**Background:**

Within healthcare, the barriers and enablers that influence clinicians’ ability to speak up are well researched. However, despite the receiver of the message being identified as a key barrier to a speaker voicing a concern, there have been very few receiver-focused studies. As a result, little is known about the barriers and enablers that influence message reception. Understanding these can help inform speaking up training and ultimately enhance patient safety through more effective clinical communication.

**Objectives:**

To identify enabling or inhibiting factors that influence the receiver’s reception and response to a speaking up message, and if the identified barriers and enablers are related to speaker or receiver characteristics.

**Design and methods:**

Twenty-two interdisciplinary simulations were video recorded and transcribed. Simulation participants formed the patient discharge team and were receivers of a speaking up message, delivered by a nurse at the patient’s bedside. How the message was delivered (verbose or abrupt wording), was manipulated and counterbalanced across the simulations. Within the post simulation debriefs, barriers and enablers of being a receiver of a message were explored using content analysis.

**Setting/participants:**

This study took place in a large Australian tertiary healthcare setting. Participants were qualified clinicians of varying disciplines and specialties.

**Results:**

A total of 261 barriers and 285 enablers were coded. Results showed that how the message was delivered (differing tone, phases, and manner) influenced what receivers identified as barriers and enablers. Additionally, the receiver’s own cognitive processes, such as making positive attributions of the speaker and attempting to build rapport and collegiality, better enabled message reception and response. Receiver behaviour was negatively impacted by listening to fix, rather than understand, and not knowing in the moment how to manage their own reactions and appropriately frame a response.

**Conclusion:**

The debriefings identified key barriers and enablers to receiving a speaking up message that differ from those previously identified for senders of the speaking up message. Current speaking up programs are predominately speaker centric. This study identified that both speaker and receiver behaviour influenced message reception. Therefore, training must place equal attention on both the speaker and receiver and be inclusive of experiential conversational rehearsal of both positive and challenging encounters.

**Supplementary Information:**

The online version contains supplementary material available at 10.1186/s41077-023-00256-1.

## Background

Speaking up is a complex social interaction with a dynamic relationship between the speaker and receiver. We define a speaking up interaction as voicing a concern that challenges the status quo [1] until a resolution is achieved [2, 3] that supports patient’s or staff’s physical and/or psychological safety [3, 4]. In this context, a resolution is a negotiated mutual agreement, such as seeking further assistance from another person to help navigate a way forward, or that both perspectives are valid, and both agree to continue as is, but now with a heightened awareness of the potential risks. The ‘act’ of speaking up, e.g. voicing of the concern, is immensely important for patient safety and to date, has been the predominant focus of training programs. Here, we propose that the focus needs to shift from the act of speaking up, to speaking up being viewed as an interaction. In this way, speaking up is more than just the initiation of voice, it is a ‘safety negotiation’ [5]. It is a mutual conversation between two (or more) speech partners, the speaker (voice initiator) and a receiver (receiving the initial speaking up message). Often in clinical practice, the act of speaking up is viewed as the job to be done, whereas it should be seen as the opening line of a conversation (not its entirety). This defines the speaking up conversation where both parties have equal accountability in negotiating a way forward. Therefore, the inclusion of specifically training the receiver within speaking up programs is vital for successful negotiation.

Healthcare speaking up has been well researched with respect to barriers and enablers to voicing a concern [6, 7]. Yet, there is paucity in the research identifying barriers and enablers to receiving the message and formulating a response that is conducive to respectful negotiation and achieving the desired shared accomplishment. We know from speaker orientated studies, that the receiver of the message is a key influential factor when deciding to speak up [6, 8]. Reception of a message has been shown to both inhibit and enhance future speaking up behaviour. For example, nurses during COVID-19 in the United Kingdom identified ‘deaf’ or hostile responses to their speaking up endeavours rendered feelings of futility and disengagement [9]. Whereas Szymczak [10] found that clinicians were more likely to speak up, if speaking up had been well received and they were thanked for their efforts. Weiss, Kolbe [11] identified how reflection on communication behaviour in after-event reviews, helped to reduce the hierarchical differences between a speaker and receiver of differing disciplines. Such work has influenced the development of speaking up training and organisational cultural programs, aiming to flatten the impact of receiver hierarchy, reduce fear of repercussions and retribution [12, 13]. Such programs routinely implement mnemonics to help speakers find confidence in the moment to initiate their voice and to structure their concern in a ‘respectful’ manner [14–16]. Despite this, speaking up remains difficult and little attention has been paid to the receiver skill set.

With few receiver-focused healthcare studies, evidence informing speaking up has been translated from other domains. Message reception has been studied in social psychology where Krenz, Burtscher [17] looked at the impact of speech content on effective team communication. They found receiver response to voice depended on how the speaker verbalised a message: using oblique, explicit, or respectful voice, and if the receiver (team leader) deemed speaker voice to be promotive or prohibitive. Within a population of business students, speaker messages were evaluated more positively when the speaker presented solutions, rather than just problems [18]. In the field of management, the speaker’s message was evaluated higher if it was perceived as supportive rather than challenging [19]. Amongst credit union employees, supervisors favoured messages from employees of higher status, than those of lower status [20]. However, these studies do not consider the complex nature of the healthcare. Most frequently, speaking up conversations occur during patient care activities where time can be critical and outcomes to these conversations can be potentially catastrophic [21, 22]. In short, the stakes can be very high.

A review of the healthcare literature revealed two receiver focused studies both conducted within the perioperative environment. Lemke, Burtscher [23] explored receiver behaviour through direct observation during anaesthetic induction using event-based behaviour coding. The authors identified four different ways receivers responded: provision of a short approval, elaboration, rejection, and non-verbal gestures, such as eye-rolling. Results showed that how the message was perceived impacted receiver responses and the likelihood of implementation of the speaker’s suggestions. Long, Jowsey [24] qualitatively analysed message response within the wider perioperative environment through interviews and focus groups. Three themes emerged: the act of speaking up, receiver filters and potential impacts of receiver’s response. They identified how the perceived negative receiver responses threatened effective teamwork by impairing working relationships or causing miscommunication due to different discipline cultural norms, e.g. how responses are phrased, blunt/direct (anaesthetist), verses non-judgemental (nurse). Long’s work informs how receiver professional identity defined by discipline, can influence message reception and response.

The current identified gaps in speaking up literature are (1) the identification and deeper understanding of the receivers’ barriers and enablers for message reception and response, (2) understanding receiver behaviour outside of the perioperative environment and across multiple healthcare disciplines. We aimed to address these gaps by identifying barriers and enablers to message reception and response within an interprofessional environment, and general inpatient ward. Simulation was used as a means to create a situation where a patient safety negotiation was required. The authors have previously studied receiver behaviour (message reception and response) in one-to-one interactions (one speaker, one receiver) [5, 25]. This study extended the knowledge of receivership and added the complexity of speaking up to an interdisciplinary team without a clearly identified receiver.

Communication Accommodation Theory (CAT) [26] posits that how a message is delivered and its perceived level of accommodation (meeting the receiver’s communication needs and level of communication competence) influences message reception, response, attributions made by the receiver and their behaviour in future interactions [27]. Within our other receiver focused studies via self-reported data, the application of CAT has helped to demonstrate how receiver reactions and responses have been consistently influenced by message delivery [25]. Therefore, we deemed it important that the speaking up messages should vary in tone, manner, and phrasing to ascertain if the level of speaker accommodation influenced receiver reactions and responses within an observed interaction (simulation). The focus of this paper therefore is the exploration of participants receiving experiences. Specifically, (1) what enabled or inhibited the receivers receiving and responding to different speaking up messages, (2) were the identified barriers and enablers to message reception related to the speaker’s, or the receiver’s characteristics?

## Methods

The authors observed and explored receiver behaviours (message reception and response) through the lens of the constructivist approach. This approach believes that there is no one truth but rather multiple realities regarding speaking up experience which are best explored through qualitative methodologies [28]. This study had full Human Research Ethics Committee approval from the Mater Health Service HREC/18/MHS/78.

### Procedure

The simulation study was conducted within a single health organisation providing both public and private health services across adult medical and surgical services, oncology, maternity, neonatal and adult critical care. Data collection occurred between May and November 2019. Purposive sampling was used to recruit qualified clinicians from varying clinical disciplines and specialties. Simulation provided a context that helped to facilitate meaningful interactions between the disciplines. All participants were clinicians attending a mandated speaking up training program. Participants self-enrolled into the program, which included simulation as a core training element. Participants then chose to either undertake the standard program (rehearsal of speaking up), or voluntarily consent and participate in the research simulation activity (receivers of a message). Both activities ran concurrently within the program. As a result, each simulation differed in size and clinical discipline. At least two nurse/midwifes were present in each simulation, medical officers were present in 11 and allied health in 10 simulations. The study consisted of 22 sessions for data collection.

The simulation was a clinical scenario where a junior nurse named Mary (embedded simulated participant) had to speak up to a multidisciplinary discharge team (the participants) during a patient bedside round. The patient was Mrs Williams (manikin voiced by a simulation technician via microphone) who had had a fall at home and was being cared for by Mary. The study participants formed the patient discharge team undertaking the round and were therefore the receivers of the nurse’s speaking up message. Upon arrival of the team to the bedside, Mary spoke up about her concern including that the patient discharge had not been completed and no home support was in place; therefore, the patient was at high risk of another fall. Moreover, the ambulance was on the way to transport the patient home and Mary did not believe the patient should go home without appropriate care in place.

Mary consistently spoke up using hint and hope methodology across all 22 simulations. Hint and hope is when one person wants another person to do/know something specific, or change behaviour, but is unwilling to address the issue directly [12, 29]. This is often perceived as helping to avoid conflict. This method of speaking is commonly used by lower status positions when voicing concerns to those of higher authority and therefore, commonly cited as the manner in which nurses frequently communicate [30–33]. For this reason, and to ensure authenticity, the hint and hope approach was chosen across both message types.

To study the impact of message delivery, two different message types informed by CAT, were designed and alternated across the 22 simulations. The nurse hinted and hoped by being theoretically accommodative (respectful and polite) and providing a lot of general information (being verbose) about the situation in eleven simulations. In the other eleven simulations, the nurse hinted and hoped by being less polite and more abrupt (less accommodative), see Table 1. If the receivers of the message (discharge team) sought further clarification of the concern, Mary the nurse would use the organisation’s safety phrase ‘I’m concerned’ which was part of the organisation’s escalation algorithm, e.g., “I'm concerned that Mrs Williams is going home by herself. I don't think that she'll be able to cope when she goes home”. After the simulation, a structured debrief was conducted and is the focus of this paper. The debriefs were conducted by facilitators trained in the debriefing methodology, Debriefing with Good Judgment [34].

**Table 1:** Example of the speaking up messages the receivers (participants) received

What Mary was thinking	What Mary actually said
The patient lives alone with no home support. She has a high fall risk. Mary is thinking the patient should not go home today due to high risk of falling. The ambulance transport needs to be cancelled and a comprehensive discharge plan organised.	Message 1: hint and hope—accommodative, verbose. 'Thank goodness, you're here. This is Mrs. Williams she's due to go home today. The ambulance is coming within two hours, but the discharge has not been organised. So, I'm really worried that the appropriate care is not going to be in place. I really think we need a decision like now, whether she can go home or not. As I said, the discharge is not in place, so wondering what your thoughts are?'
	Message 2: hint and hope—less accommodative, abrupt.' Look, I asked the ward receptionist if you could start the ward round here, not finish here. I've got the ambulance transport coming to pick up Mrs. Williams and none of the discharge has been done and I don't think she's ready to go home. So, I just need you to do something.'

Prior to data collection, debriefers attended an onboarding session outlining research simulations and objectives. All debriefers had previously undertaken formal debriefing training. For the first round of data collection, faculty worked in pairs to help ensure a consistent approach to the debriefing process. This was supported by an Objective-Orientated-Debriefing tool, to help structure and guide the debriefing process [35]. The faculty used open-ended questions followed closely with further inquiry; why and how questions (advocacy/inquiry) [34]. This approach helped to obtain more in-depth perspectives of the participant’s experiences within the simulation [36], both as an individual, and collectively as a group who just had shared a speaking up encounter. At the completion of data collection (22 debriefs), the faculty’s performance was evaluated via the Debriefing Assessment for Simulation in Healthcare (DASH) tool [37] to ascertain consistency between debriefers.

### Data collection

All the debriefs were video recorded by both Go-Pro camera and the simulation centres inbuilt audio-visual (AV) system to mitigate the risk of audio-visual recording failure. Video data were stored on a password protected external hard drive and then uploaded into a research data management system. All 22 debrief videos were reviewed for audio quality and were included for analysis. The debriefs were transcribed verbatim using NVivo transcription [38] and cross checked with the video recordings for accuracy.

### Data analysis

Coding of the barriers and enablers for receiving the speaking up message were inductively analysed and deductively categorised using a content analysis approach [39]. Firstly, coders (MB, KM) met and discussed the approach to data analysis and to reach a general shared understanding of the data. The data were then read through three times by the lead author (MB) with reflexive notes made on each iteration to immerse self in the data. MB then inductively generated the initial codes, creating a code sheet. Using the initial codes, all 22 debriefs were then independently deductively analysed by the two coders (MB, KM). Through discussion, codes and definitions were refined. MB reanalysed all the data according to the new coding scheme (see supplementary document). Using the revised code sheet, KM then randomly chose and double coded 7 (30%) debriefs out of a hat, for consensus and sound interpretation of the data [40]. All meetings between coders were recorded as part of the reflexive journaling process. Interclass correlation coefficient (ICC) for absolute agreement to ascertain interrater reliability was calculated against the 30% double coded data, using mean-ranking, 2-way mixed effects model [41]. Codes were then deductively grouped under four categories aligning with research previously undertaken in healthcare speaking up, from the speaker’s perspective [6]: ‘climate’, referred to concepts such as organisational culture, speaking up and receiving methods; ‘relational’ for example, was how the receiver perceived the speaker and the relational dynamics within the interaction; ‘self’, referred to the receiver’s level of self-awareness and how this influenced their behaviour, and ‘content’, was the message itself and the manner in which it was delivered. On completion of double coding and consensus, frequencies of codes for receiving a speaking up message were calculated. Frequencies of barrier and enabler codes are reported separately and reported as a percentage of the total codes to help identify trends.

Both coders were Caucasian, female registered nurses with an extensive history in healthcare education and simulation and had experience in developing and delivering speaking up training programs. Author MB acknowledges that prior personal confronting experiences with receivers of speaking up messages may have influenced the qualitative analysis process. One in particular, resulted in patient harm [42]. Author KM has encountered multiple speaking episodes over 40 years of experience and has been on both sides of the conversation as sender and receiver. KM acknowledges that receiving speaking up is more difficult than being the speaker. This experience has fuelled a deep interest in understanding and creating programs to help develop receiver skills.

DASH ratings were completed on the 22 video recorded debriefings by the two researchers (MB & KM). The DASH utilises a 1 to 7 scale and has undergone prior validity and reliability testing [43]. The two raters were both formally trained in debriefing methodologies and certified raters in the DASH tool. Both reviewers reviewed two debriefs together then independently reviewed the subsequent debriefs, coming back together every fifth listed debrief for consensus. Inter-rater reliability of reviewer scores was assessed via both adjacent agreement (%), and exact agreement via Cohen’s kappa.

## Results

Twenty-two debriefs were analysed, 11 with accommodative/verbose wording and 11 with less accommodating, abrupt wording. The average debrief time was 14:37 min (range 10:0–20:10 min). There were 138 clinicians who participated in the debriefing sessions. Nurses/midwives [NM] (*n* = 96), allied health, [AH] comprising of social workers, physiotherapists, radiographers, pharmacists, and phlebotomists (*n* = 22) and medical officers [MO] (doctors) (*n* = 20), refer to Table 2 for participant characteristics. A high degree of reliability was found between the two raters on the 7 double coded simulations (30%). The average ICC was 0.988 with a 95% confidence interval from 0.975–0.994. For the assessment of the debriefers using the DASH, inter-rater reliability was assessed via adjacent agreement of ± 1 scale point of the 7-point scale, resulting in an adjacent agreement of 100%. Exact agreement was calculated via Kappa = 0.56.

**Table 2:** Participant characteristics (*n* = 138)

Characteristic	Nurse/midwife (NM)		Allied health (AH)		Medical officer (MO)	
	*n*	%	*n*	%	*n*	%
	96	69.5	22	16.0	20	14.5
*Clinical specialty*						
Critical care	18	18.8	1	4.5	7	35
Perioperative	13	13.5	0	0	2	10
Inpatient Wards	35	36.5	8	36.4	5	25
Day stay areas	1	1.0	0	0	0	0
Antenatal areas	2	2.1	3	13.6	0	0
Birth suite	7	7.3	0	0	2	10
Outpatients	4	4.2	4	18.2	2	10
Interventional areas	5	5.2	3	13.6	0	0
Other	8	8.3	2	9.1	2	10
Missing	3	3.1	1	4.5	0	0
*Years in profession*						
3 years or less	29	30.2	5	22.7	10	50
4 to 8 years	22	22.9	7	31.8	2	10
9 to 14 years	11	11.5	4	18.2	5	25
15 to 20 years	7	7.3	3	13.6	1	5
More than 20 years	11	11.5	2	9.1	2	10
Missing	16	16.7	1	4.5	0	0
*Gender*						
Male	11	11.5	6	27.3	10	50
Female	85	88.5	16	72.7	10	50

The debriefing transcripts were coded, and frequencies of codes obtained, see Table 3. By chance, there were 69 (*n* = 69) participants in each of the accommodative, verbose, and less accommodative, abrupt simulations (total *n* = 138).

**Table 3:** Frequency of codes per category for barriers and enablers to message reception

Accommodating verbose speaking up		Abrupt speaking up	
Enablers	Frequency	Enablers	Frequency
Content	57	Content	6
Relational	66	Relational	86
Self	19	Self	35
Climate	8	Climate	8
Total	150	Total	135
Barriers		Barriers	
Content	31	Content	36
Relational	19	Relational	31
Self	42	Self	58
Climate	14	Climate	30
Total	106	Total	155

**Fig. 1:**
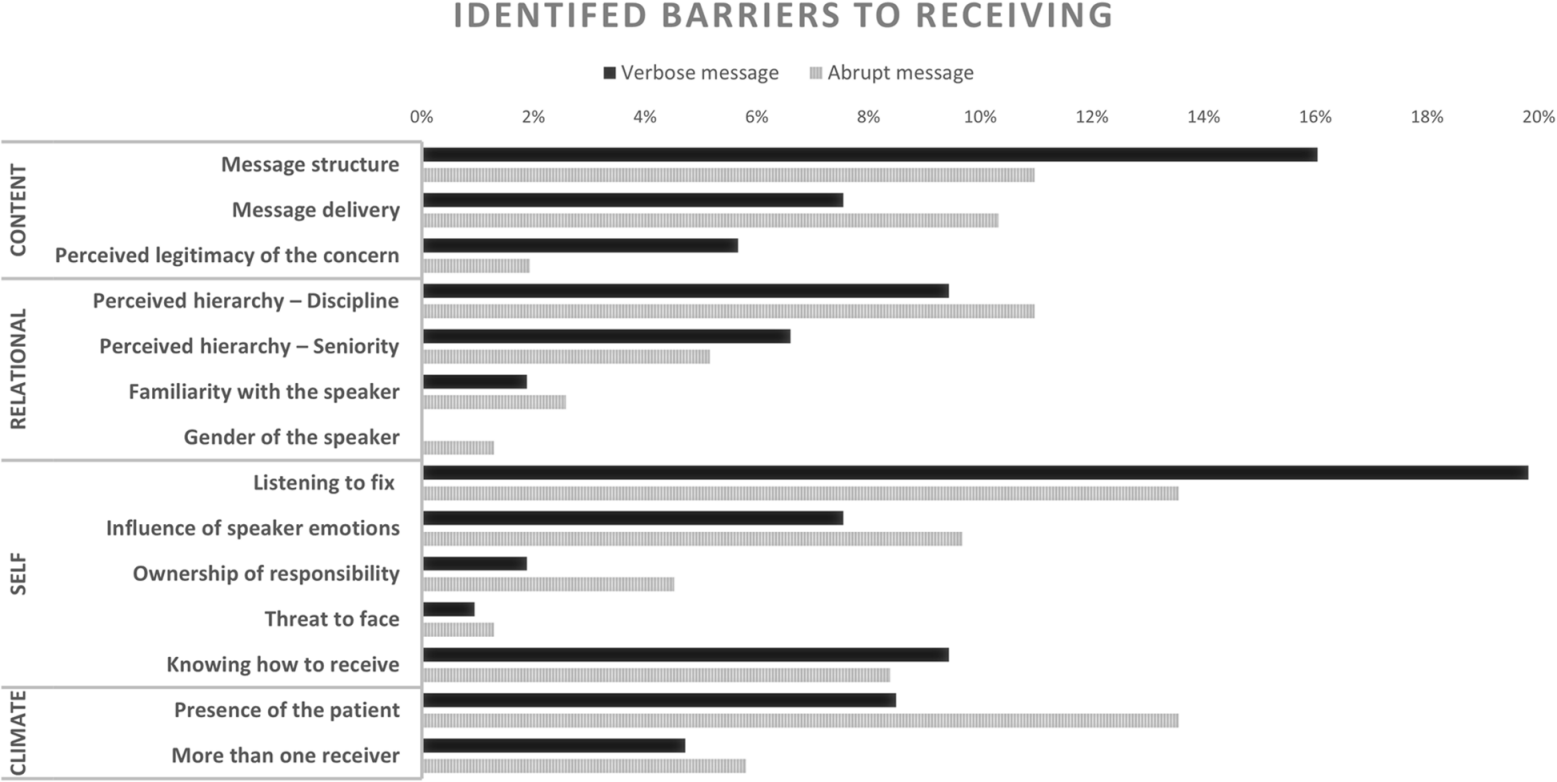
Barriers to receiving a speaking up message described during post simulation debriefs of 138 multidisciplinary participants

**Fig. 2:**
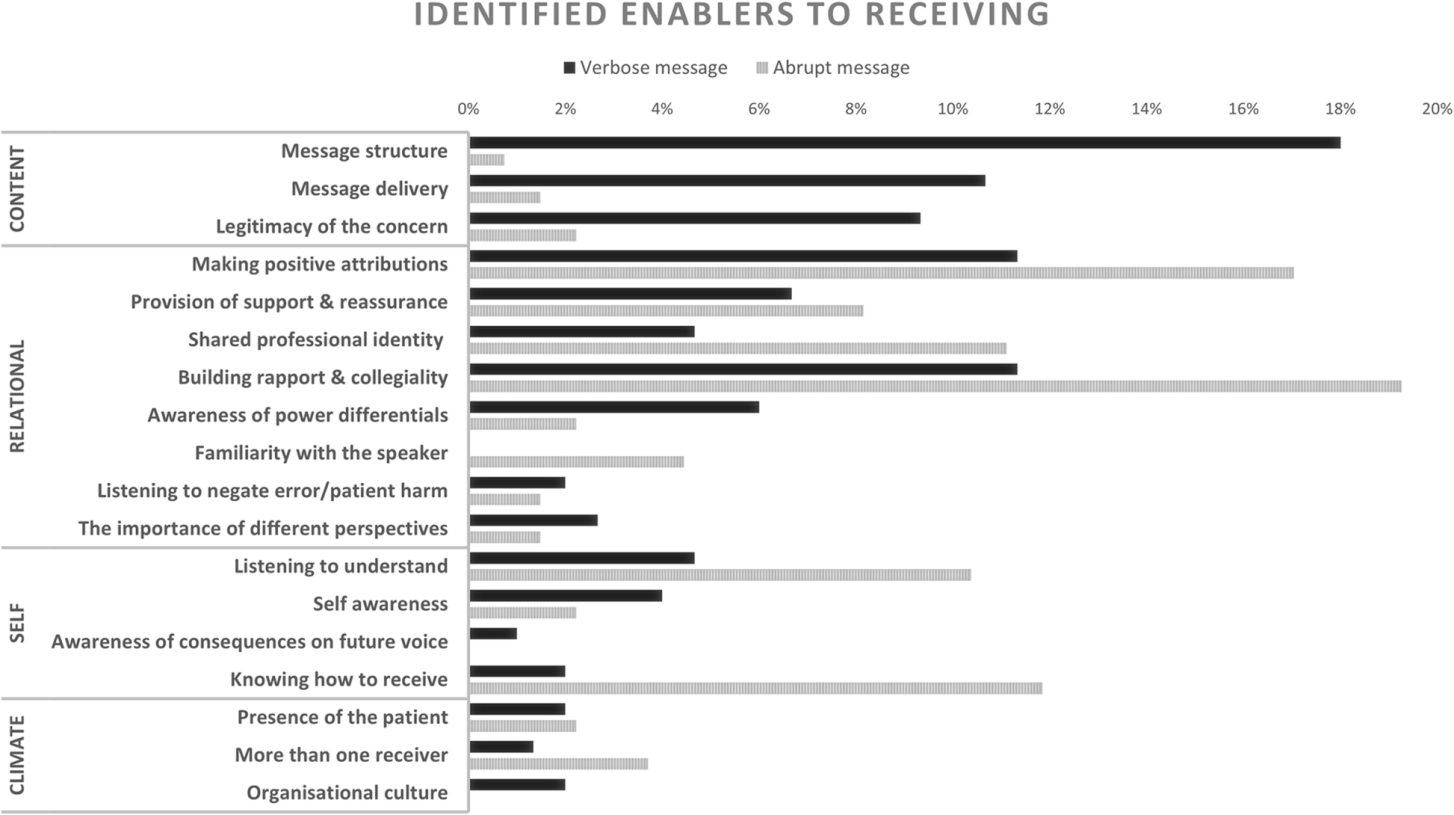
Enablers to receiving a speaking up message described during post-simulation debriefs of 138 multidisciplinary participants

Each code was presented as the percentage of the total codes for both barriers and enablers, see Figs. 1 and 2. For the more accommodative but verbose speaking up message, the four most identified barriers to message reception were listening to fix (21[20%]), message structure (17, [16%]), knowing how to receive (10, [9%]), and equally perceived hierarchy of the speaker as defined by clinical discipline (10, [9%]). Within the simulations where the message delivery was more abrupt, the four most identified barriers were listening to fix (21, [14%]), the presence of the patient (21, [14%]), perceived hierarchy of the speaker as defined by clinical discipline (17, [11%]), and message structure (17, [11%]).

Listening to fix referred to receivers listening to identify tasks to be done, rather than listening to understand the concern being raised, and was identified as the most frequent theme across both message types.

“I guess you get that tunnel vision, you get so focused on the task that we had were there to discharge a patient that afternoon that it took the repetition of her saying three times before I actually stopped and took a second to realize, okay, I need to address something here” (NM 42).

Message structure is related to the clarity of the concern and the phrasing used by the speaker. Receivers described that the message was either not clear or direct enough, e.g. “I think just a bit more direct about what you need. I think it's very hard to pick out what she was actually wanting us to do” (NM63), or the message only contained the problem and no solutions or recommendations “The nurse couldn't show any solutions” (AH07). The more accommodative and ‘polite’ messaging was for many seen as a barrier, as speaking up is not normally framed as a ‘polite’ conversation, e.g., “We haven't really practiced it in a positive way. It's always a matter of disagreement or trying to approach something that's bad that's happened” (NM09). Knowing how to receive a non-confrontational message was an identified barrier, “I think I was quite confused. Because I think I was expecting a complication. Just didn't really know what the crux of it was” (NM10).

The conversation occurring in the presence of the patient, influenced receiver responses and the evaluation of the situation, particularly with the more abrupt messaging, e.g,. “I did feel like we should not have been having that conversation in front of the patient” (AH 04). The speaker’s discipline and the perceived hierarchy of that discipline in relation to the receiver’s own, was described as impeding receiver voice and their ability to effectively engage. Speaker discipline was predominately described by receivers as apprehension to receive messages and communicate with medical officers within real clinical practice.

“If they're like a doctor or someone, I'm just gonna [sic] cop it. I'm just going to say, okay, I'll agree with you. You know, unless it's something that is really completely unsafe to the patient, then you have to” (NM45).

Nursing, midwifery, and allied health participants did not describe a medical officer speaking up as speaking up, rather it was perceived as a nonnegotiable order. See Supplementary data Table S1 for coding definitions and exemplar quotes for each barrier.

There was a larger distribution of identified enablers compared to barriers. For the more accommodative/verbose speaking up message, the four most identified enablers were message structure (27, [18%]), making positive attributions (17, [11%]), building rapport and collegiality (17, [11%]), and message delivery (16, [11%]). Within the simulations where the message delivery was less accommodative and more abrupt, the four most identified enablers were building rapport and collegiality (26, [19%]), making positive attributions (23, [17%]), knowing how to receive (16, [12%]), and shared professional identity (15, [11%]).

Receivers described message structure as an enabler only within the more accommodative and verbose speaking up messages. Receivers described that the message was clear and easily interpreted, e.g. “I think she was concerned she was going to cope at home. She communicated that very clearly, I thought.” (MO05), and/or the speaker used a known safety phrase ‘I’m concerned’ that made the receiver pay more attention to the speaker.

“When she used those key words, I'm concerned, and she was able to list off her concerns. And then using the word 'safe' where you say, I'm concerned about the safety of my patient, when you use particular keywords, that makes you think a little bit differently and you take it a lot more seriously” (NM42).

Message delivery, that is the way the message was delivered, was only described as an enabler again, with the more accommodative and verbose speaker message.

“Yeah. I think also with the tone and stuff, my mood almost like made me feel a bit more respected… if someone's yelling at you, I feel like they're not respecting you very much. But if they're being calm and like talking to you, then there's a bit more of respect going back and forth (AH03).

Building rapport and collegiality, e.g., the conversation was, or aimed to be collaborative, through building rapport and/or perceiving equal accountability, “It felt like we were all working together” (NM08), and “it's more like asking, do you think this is the right thing? Like, you know, I'm not really sure myself, like maybe let's think about this together, more than, I'm right and you are wrong” (MO02).

Making positive attributions about the speaker related to the receiver giving the speaker the benefit of the doubt and/or holding them in positive regard, regardless of how speaker behaviour was perceived. Receivers described making more positive attributions about speaker intent with the less accommodating/abrupt messaging.

“I think just understanding. Like others, you know, we are all busy and if things aren't done the way you want them to be done, or in the time you want them to be done in, there is often a good reason” (MO10).

Receivers described when spoken up to with the more abrupt wording, that knowing how to receive and respond to a message was important to have an effective conversation. One receiver described that instead of getting defensive to the message instead say “I'm thinking that you're really worried about this patient. I'm wondering what I can do for you?” (NM26).

Sharing a professional (social) identity (e.g., nurse speaker, nurse receiver) enabled receivers to align themselves with the speaker and internally empathise with the speaker’s situation; “It's easier to hash it out with someone if you're a similar job role, because you both understand the minute problems you face, like so you kind of both understand what delaying someone's discharge will mean to this and that” (NM76).

See Supplementary data Table S2 for coding definitions and exemplar quotes for each enabler.

## Discussion

This study sought to identify barriers and enablers of receiving speaking up messages by undertaking a qualitative analysis of 22 debriefs. We found that the characteristics of message delivery (differing tone, phases, and manner) influenced what receivers identified as barriers and enablers. Additionally, the receiver’s own cognitive processes, such as making positive attributions of the speaker and attempting to build rapport and collegiality, better enabled receiver responses.

Overall, there were a greater number of enablers (predominately Relational) identified than barriers (predominately Self). However, the distribution and frequency of the enablers were more dispersed. This could indicate that what helps receivers to hear and process the message, is harder to define than the barriers. It may be that receivers genuinely do not have clarity as to what enables them to effectively listen, process the message, and frame a response. Not knowing how to receive the message was identified as a key barrier, and the prospect of being trained how to receive a message, was perceived as an important enabler.

The first research question was to identify enabling and inhibiting factors for receivers when engaging in a speaking up conversation. Results demonstrate that the ability of the receiver to make positive attributions, or hold ‘The Basic Assumption’ [44] of the speaker, is an important skill for receivers to develop. The ability in the moment to hold the speaker in positive regard, regardless of how the message was delivered, helped receivers engage in the conversation and is in alignment with our previous study, specifically within the allied health receiver group [5]. Positive attributions, paired with the desire to build rapport and collegiality with the speaker, were particularly noted when the speaker was less accommodative, more abrupt. These results build on the work of Weiss, Kolbe [45] who examined the behavioural effects of agency and communion. Applying their findings to our results, we can theorise that when receivers deployed rapport building tactics, they were trying to not just modulate their own reaction, but also enhance communion (helpfulness) behaviour. This behaviour supports positioning oneself to be more curious, to further understand the speaker’s perspective, and for the receiver to respectfully share their perspective (agency) to help move the conversation forward.

This desire to be, or appear to be collegial, may have been due to the presence of the patient. The patient’s presence was a clear barrier for receivers across both message types. Unlike feedback conversations, speaking up most frequently occurs, and needs to occur, during clinical practice where others are present. Schwappach and Gehring [32] found that the presence of the patient inhibited speaking up, mostly due to the speaker’s desire not to humiliate a colleague in public or lose patient trust. Our work across multiple receiver focused studies has found that indeed, the presence of an audience consistently influences the receiver’s reactions and responses, particularly nurses and midwives and when the speaker uses less accommodative language [25, 46]. Clearly, to enhance message reception, both the speaker and receiver need to negotiate not just the raised concern, but also the context in which the conversation occurs (in nonlife-threatening situations). Predominately, this is not routinely taught, or tangibly addressed within speaking up programs.

Interestingly, regardless of how the message was delivered, receivers described an overall unpreparedness to receive and respond when spoken up to, specifically what to say, and how to clarify further to help their understanding. This resulted in extrapolated conversations and a delay in understanding the problem [46]. Bodie and Villaume [47] and Ayres, Wilcox [48] described the phenomena of receiver apprehension, referring to the anxiety elicited when the receiver feels fear and inadequacy of understanding and processing the information in high demand, high cognitive load situations. This was displayed by many nurses/midwives and a few allied health, who defaulted to ‘fixing’ (listening to fix). Nurses/midwives wanted to quickly ascertain what tasks could be done, even if these were not directly related to the main concern of the speaker. These receivers described wanting to be helpful to the speaker’s needs and be prompt in resolving the problem, even though they weren’t clear yet what the actual problem was.

What may help, is for speaking up mnemonics to include recommendations or desired outcomes the speaker is seeking. Receivers often discussed frustration as the speaker didn’t offer solutions and recommendations when raising the concern, often referring to the handover tool ISBAR [49] as a point of reference (R stands for recommendations). Although this finding is in alignment with other work [50], we found the result surprising, as speaking up mnemonics don’t typically include speaker recommendations for next steps, e.g. PACE (Probe, Alert, Challenge, Emergency) [51], CUSS (I am Concerned, I am Uncomfortable, This is a Safety issue and Stop!) [52]. Although ISBAR has been endorsed as an escalation tool for deteriorating patients [53], it is routinely cited as a clinical handover tool [54]. This finding builds on organisational literature on voice, where speakers (voicer) who suggest solutions rather than problems, were viewed more favourably and received higher performance evaluations [18]. Whiting, Maynes [18] suggested future studies need to ascertain how group membership of the voicer (speaker) and rater (receiver), could possibly influence these outcomes. Our study confirms that speaking up conversations within the health context are strongly influenced by group membership, as defined by clinical discipline and seniority [25]. We recommend further research needs to be undertaken to see if the inclusion of recommendations as a generic and standard speaking up process enhances or inhibits message reception, and in what context (low or high stakes situations).

The second research question aimed to understand if the identified barriers and enablers were due to characteristics of the speaker, or the receiver. Fundamentally, it was both. The barriers and enablers show that there is equal accountability on the speaker and receiver for the conversation to be successful. What was unusual, was that receivers in the more accommodative, verbose messaging described message structure as both a key enabler and barrier, whereas the more abrupt messaging (structure and delivery) was only identified as a barrier.

The accommodative, verbose message was an enabler due to the manner of the speaker being evaluated as polite and the concern being more legitimate. Where this messaging was seen as a barrier, receivers explained that the general narrative around speaking up in healthcare is that it is framed as a ‘difficult conversation’, and training programs discuss and rehearse more confrontational examples and situations. By doing so, receivers were unsure how to respond when the interaction was not confrontational. Vauk, Seelandt [55] also reported this paradox from the speaker’s perspective. Civil receiver behaviour increased the difficulty to speak up, compared to uncivil behaviour. The authors thought this may be due to the perceived risks of damaging interpersonal relationships, or incivility triggering anger, lending the speaker to find their voice. Within our study, receivers described the ‘non-confrontational’ message influenced their evaluation of the legitimacy, and/or level of urgency of the concern. This caused confusion, influencing how they initially reacted and responded, and their comprehension that it was a ‘speaking up’ conversation. This potentially led to receiver inaction. We suspect that the polite messaging diluted the seriousness of the problem for these receivers. Speaking up that is evaluated as non-confrontational, and an ‘easy conversation’, may be less impactful on the receiver, and influence the receiver’s evaluation of the level of attention and urgency required.

How the message was delivered had impact. The seemingly ‘politer’ hint and hope messaging adopted by nurses, has shown to cause frustration for medical officer receivers, influencing the sense of situational urgency and increased cognitive burden [31]. Within our study, nurse/midwife receivers described being frustrated by the indirect nature of the message. This led to some significant insights into how their indirectness when speaking up in real clinical practice, makes it so much harder for the receiver to understand their concern and ascertain their conversational goals. This finding does support the use of conversational mnemonics to help speakers frame a succinct message. Previous studies demonstrated how the use of advocacy/inquiry was evaluated by receivers as an appropriate methodology to frame a concern, provide a rationale, and seek the receiver’s perspective [5]. Lemke, Burtscher [23], however, found that while advocacy/inquiry resulted in more conversations between speaker and receiver, there was greater receiver inaction of the speaker’s suggestions. Clearly, more work needs to be undertaken to understand framing of speaking up messages from the receiver’s perspective, highlighting the complexity of these interactions.

Along with the messaging, receivers discussed the negative impact of the speaker’s emotions. Receivers described and evaluated the speaker as being stressed, frustrated and anxious which directly impacted their own behavioural reactions. Receiver’s not knowing how to receive, not just what to say, but how to manage their own internal reactions, were key identified barriers. Schein and Schein [56] describes an individual’s verbal response and their demeanour is highly dependent on their internal cognitive processes. Attributing positive intentions allows for a more positive evaluation of the speaker, which helps to achieve a curious mindset for deeper understanding of the speaker’s problem [56, 57]. However, this can only truly be achieved if receivers are able to acknowledge the speaker’s emotional needs, and manage and/or reframe their own reactions to enhance listening and comprehension [58]. This is essential if they are to meaningfully participate in a shared negotiation [5]. This requires equipping receivers with strategies to achieve this, alongside deliberate practice and ongoing reflection and feedback. As identified in previous studies [45, 59], debriefing is an ideal tool to facilitate interprofessional reflective and reframing discussions, to help clinicians understand differing perspectives and discipline specific barriers and enablers.

When the conversation was generalised to real clinical practice, receivers identified speaker discipline impacted their reception of a message. Nurses, midwives, and allied health described that they did not define a medical officer raising a concern as speaking up, rather it was a non-negotiable order. This finding was supported in a recent article describing real speaking up encounters. To enhance interdisciplinary receiver response, medical officers need to clearly verbalise when they are speaking up, and actively invite receiver input. This invitation to speak, has shown to enhance junior speaking up behaviour [60] and needs to be applied for more junior, or traditionally lower hierarchical receivers.

### Limitations of the study

Limitations of the study included the simulated environment. To offset this, substantial effort was made to design and deliver the highest quality of simulation in consideration of creating an authentic experience and by aligning to best practice standards and evidence. Additionally, all debriefers were trained in the same methodology of debriefing and demonstrated consistent, high-quality debriefing, as supported by DASH results. Secondly, the participants still knew they were being observed, and participation in an interdisciplinary round may not be part of their normal clinical role, potentially impacting their ability to fully engage. Data collection occurred at a single site and although participation in the receiver focused simulation was voluntary, we acknowledge that attendance to the speaking up program was not. Finally, the clinical event simulated in the scenario was a not an occasion where patient harm was imminent, and therefore caution extrapolating the results to high stress, high emergent situations need to be taken into consideration.

## Conclusion

Simulation debriefs identified key barriers and enablers to receiving a speaking up message within the context of a patient discharge with an interdisciplinary team. Findings demonstrated that both speaker and receiver behaviour influenced message reception. The results indicated that speaking up programs need to include training for receivers: to help shift from listening to reply or fix, to listen to understand, manage both the speaker’s and their own emotions in the moment, and view speaking up as a shared accomplishment. To do this, educational programs must train the speaker and receiver equally through conversational rehearsal of both positive and challenging encounters. Simulation is uniquely positioned to provide the learning space for this training.

## Supplementary Information

**Additional file 1: Supplementary data Table S1.** Codes, Definitions and Examples of the Barriers to Receiving a Speaking Up Message.** Supplementary data Table S2.** Codes, Definitions and Examples of the Enablers to Receiving a Speaking Up Message.

## Data Availability

All data generated or analysed during this study are included in this published article and its supplementary files.

## Abbreviations

NM: Nurse/Midwife
AH: Allied Health
MO: Medical Officer
DASH: Debriefing Assessment for Simulation in Healthcare
