# Supplementary material for: Identification of the barriers and enablers for receiving a speaking up message: a content analysis approach
Source: Adv Simul (Lond). 2023 Jul 6;8:17. doi: 10.1186/s41077-023-00256-1 (PMC10324134; doi:10.1186/s41077-023-00256-1)
Supplement: Supplementary file 1 — Additional file 1: Supplementary data Table S1. Codes, Definitions and Examples of the Barriers to Receiving a Speaking Up Message. Supplementary data Table S2. Codes, Definitions and Examples of the Enablers to Receiving a Speaking Up Message. [file 41077_2023_256_MOESM1_ESM.docx]

# Supplementary Data Table 1

Codes, Definitions and Examples of the Barriers to Receiving a Speaking Up Message

| **Code** | **Definition** | **Examples** |
| --- | --- | --- |
| **BARRIER - Content** | | |
| Message structure | Receiver feels that the speaking up message was:   - not direct enough - not clear and not easily interpreted   Message is problem focused. Speaker does not offer any solutions or recommendations. | “I think just a bit more direct about what you need. I think it's very hard to pick out what she was actually wanting us to do.” NM63  “I think that is the lesson I take to stand back on the other side and say, I really don't know what you want me to do. Try to draw out of Mary, like what actually you want me to do as standing as the medical people standing at the end of the bed. And it's for us, it's like, okay, I can just say this is what we need. Can you guys organise that or, you know, being a little bit more direct and not being embarrassed or scared of doing that like, I actually do need this and I did need you guys to do this this morning.” NM63  “The nurse couldn't show any solutions.” AH07  “It would have been really important earlier that you are the one who's been on the ward, what do you think should happen?” NM87 |
| Message delivery | Receivers feel the speaker delivers the message in manner that makes them defensive e.g., accusatory, passive aggressive.  Alternatively,  The message is delivered in a non-confrontational, respectful manner which receivers are unprepared for e.g., speaking up is normally viewed as confrontational/conflict situation, which negatively impacts their ability to respond. | “I think it shows a bit of a deer in headlights moment for everyone because I did not expect her to go off like that, actually.” AH14  “I think it was sort of one of those situations where everyone's aware that she wasn’t being quite professional. And, it shouldn’t have been said the way it was said.” NM48  “I was sort of expecting Mrs. Williams to have an argument about not being discharged… just going from everything that we've been taught [in speaking up], I guess it was more in terms of arguments.” NM53  “We haven't really practiced it in a positive way. It's always a matter of disagreement or trying to approach something that's bad that's happened.” NM09 |
| Perceived legitimacy of the concern | Receivers do not share the same level of concern for the raised problem, or question the legitimacy of the concern based on who the speaker is.  Or,  Receivers feel it is not their role to fix the problem. | “I thought they were [speaker’s concerns] trivial at first.” MO06  “I have had that before where we've had such a scenario where a dietician was concerned about something that was happening. And at first you do take it on board of like, I guess not acknowledging them. I think my communication is poor sometimes like not taking their concerns as seriously as what they're taking it.” NM27 |
| **BARRIER - Relational** | | |
| Familiarity with the speaker | Familiarity impedes speaking up communication: seen prior poor behaviour as a bystander, &/or had previous experience with poor speaking up encounters.  Or, the receiver is completely unfamiliar with the speaker and does not know how they will react or engage | “Certainly, I've had to deal with consultants all over the place. If you have a poor experience with the consultant, one particular consultant, it makes it much harder to carry on to replicate it again.” AH10  “It's hard when you don't know the people.” AH02 |
| Perceived hierarchy: Seniority | The seniority (years of experience and level) impedes receiver’s ability to effectively engage in the conversation | “For some of my colleagues it would be offensive for a junior person to dare and say something.” MO08  “Personally, I’m also a junior nurse and I find it more difficult to speak up to people within my own profession because I'm interacting with them so often. I don't want to get on their bad side. I know you're working with them tomorrow.: NM47 |
| Perceived hierarchy: Discipline | The profession of the speaker impedes receiver’s ability to effectively engage in the conversation  Hierarchy trumps relationships. | “I agree, if it was a nurse unit manager tapped me on the shoulder and said this what you need to do, then I'd go...OK!” MO15  “The assumption is always like the doctor is always right.” AH13  “If they're like a doctor or someone, I'm just gonna cop it. I'm just going to say, okay, I'll agree with you. You know, unless it's something that is really completely unsafe to the patient, then you have to.” NM45 |
| Gender of the speaker | The gender of the speaker impedes receiver’s ability to effectively engage in the conversation. | “I find it easier to tell a male doctor when I think they are doing something wrong than a female doctor. I don’t k now if it’s because a lot of female doctors I know are a bit more assertive.” NM48  “I would probably say I find it hard to speak to other women. I don't know why. I just find it easier to speak to to speak up to men than women. Maybe because I know women can be more emotional.” NM45 |
| **BARRIER - Self** | | |
| Influence of speaker emotions | Speaker emotions:   - distract from effectively receiving the message - influences receiver’s behaviour (anxiety/stress/level of urgency) - increases receiver’s cognitive load, having to deal with both the emotions and the problem - impacts response as receivers don’t know how to manage speaker emotions. | “I think she was going a mile a minute and was trying to say everything was time critical, I immediately respond to that kind of anxious energy. I'd go immediately [flustered] Oh ok I'll help you with a physio assessment!!” AH18  “I was receiving them as this is so urgent that the normal building rapport interaction has been pushed to the side in order to manage this urgently. AH17  “We had two jobs. We had to take care of the patient and we had to take care of the nurse.” MO14 |
| Ownership of responsibility | A belief that being a receiver means:   - needing to be the expert, and having all the answers. - The concern can only be effectively addressed if it is in alignment with the receiver’s area of expertise - taking on the speaker’s problems. | “My biggest problem is myself, because my personality type, as described by people close to me is I’m a martyr. I would rather I have all the problems on my shoulders rather than anyone else's. I don’t want to burden anyone with what I perceive to be a problem or a complaint.” NM47  “I think my initial like my primal reaction was we didn't get the message, it wasn't our fault! Like, my immediate was like defence and reflect blame.” NM73 |
| Listening to fix | Receivers focus on tasks to do to ‘fix’ the problem, rather than truly clarifying, or understanding the problem.  Results in receiver not hearing or understanding the main concern, instead just looking for what can be quickly fixed. | “It’s like, just do it [tasks], it’s fine. That generally smooths things over a lot quicker.” NM79  “I guess you get that tunnel vision, you get so focused on the task that we had were there to discharge a patient that afternoon that it took the repetition of her saying three times before I actually stopped and took a second to realise, okay, I need to address something here.” NM42  “And when I really think about it, I could have listened better and that might have helped me work through that process with her. Because you said that she went through that escalation process, but I don’t remember hearing that.” NM08 |
| Threat to face | Receivers worry about how others will judge them e.g., the speaker pointing out an error is pointing out the receiver’s personal failure, leading to defensive behaviour and blame shifting. | “You feel that that’s a reflection on your capability.” NM45  “There’s nothing worse than getting on the phone and they are asking lots of questions, you don’t know the answers to.” AH10 |
| Knowing how to receive | Receivers do not know how to respond when spoken up to:   - knowing/realising you are being spoken up to words/phrases to use - how to manage receiver emotions - how to manage speaker emotions - how to engage in a conversation when don’t yet fully understand the concern - how to listen and make a decision under pressure, or with an audience present. | “I know what I want to get out of here, but I don’t know if I should say stop don’t talk to me like that.” MO16  “I could tell she was stressed but I didn’t know what to do about her stress. What happens from here?” NM78   “I just thought it was an awkward conversation because I felt I couldn’t help her.” AH12 |
| **BARRIER – Climate** | | |
| Presence of the patient | The presence of the patient impedes message reception and response. | “I did feel like we should not have been having that conversation in front of the patient.” AH04  “The patient hearing that just wasn't right.” NM18  “If I was the patient and heard it I would be thinking get me out of here!” NM20 |
| More than one receiver | Having more than one receiver impedes reception and response to the message due to:   - poor role clarity - knowing who should receive/speak first - not wanting all to speak at once, but not wanting silence either | “Do you use your role, or not as there is six of us in the team, it influenced communication in the team.” MO05  “Someone had to say something.” AH02  “I didn’t want to just jump in.” NM46 |

# Supplementary Data Table 2

Codes, Definitions and Examples of the Enablers to Receiving a Speaking Up Message

| **Code** | **Definition** | **Examples** |
| --- | --- | --- |
| **ENABLER - Content** | | |
| Message structure | Receiver feels that the speaking up message was:   - clear and easily interpreted - it was clear they were being spoken up to.   Speaking up more than once is viewed as important. Speaker using a standard phrase e.g., ‘I’m concerned’, makes receiver pay attention and think ‘what am I missing?’ | “I think she was concerned she was going to cope at home. She communicated that very clearly, I thought.” MO05  “The nurse was clearly speaking up and trying to be heard.” AH08  “When she used those key words ‘I'm concerned’ and she was able to list off her concerns. And then using the word 'safe' where you say, I'm concerned about the safety of my patient, you use particular keywords that makes you think a little bit differently and you take it a lot more seriously.” NM42  “She kept emphasising it, but she kept elaborating on why she was concerned. Yeah. I guess that was the moment I was like, okay. She keeps bringing up that she's concerned. I need to realise that, you know, we need to do more.” NM43  “If somebody says I'm concerned you've got a hundred percent of my attention.” NM22 |
| Message delivery | Receivers feel the speaker delivers the message in a non-confrontational and respectful manner e.g., tone of voice, body language | “I didn't feel it as threatening. I didn't feel like she was blaming us to try to get the patient out.” MO02  “If people speak to you in a respectful way and are kind in the delivery of their message, it's definitely easier to receive” NM27  “Just the tone, just the way people are saying where it's like, they're not blaming you. That is like, yeah, it's more like asking like, do you think this is the right thing?” MO02 |
| Legitimacy of the concern | When the nurse spoke up, the concern is deemed legitimate as nurses/midwives spend the most time with the patient.  If a nurse/midwife speaks up, you have to listen.  The concern is taken more seriously if the speaker has tried to resolve the problem themselves prior to speaking up. | “From spending eight hours as you would know [points to NM54] it's like, you know, eight hours with the patient versus two minutes. Yes, you get a very different perspective on how this patient is going and you see things that other people wouldn't get to see.” MO07  “She obviously has been looking after this patient and she knows her quite well, she knows her limitations. She's the person that we need to chat to about where we go from here.” NM63  “There was a very legitimate concern around the daughter being two days away, Blue Care not being there in time.” AH18 |
| **ENABLER- Relational** | | |
| Familiarity with the speaker | Familiarity enhances speaking up communication when:   - previous positive experiences when speaking up to the same person. - have a positive working relationship with the person - Alternatively, not knowing the other person allows greater freedom to converse. There are no preconceptions or expectations. | “I think sometimes a barrier can be preconceived or set ideas about the person involved in the incidents. So, I mean this separate to whether you have a relationship with them professionally, but I think if their reputation is one of being approachable, an excellent leader (another participant - or the opposite), you are far more comfortable to go and say I've got a problem based on my own experience.” NM49 |
| Making positive attributions | The speaker is viewed as critical in helping to find solution.  Attributing speaker intent to patient safety, or wanting to do the right thing for the patient:   - allows receivers to overlook poor speaking up behaviour - helps to engage in the conversation despite previous bad speaking up encounters - overcomes hierarchical differences (cost/benefit analysis) - focuses on the process/system, not the person. | “She wanted to do the best thing and she was worried about Mrs Williams falling and not having the right stuff.” MO03  “It was good that she advocated for her patient.” NM89  “We could have asked her, well that may not have been her only patient. If it was a heavy ward, she would have had five or six patients as well.” NM75  “I think just understanding. Like others, you know, we are all busy and if things aren't done the way you want them to be done, or in the time you want them to be done in, there is often a good reason.” MO10 |
| Provision of support and reassurance | Speaker is speaking up in seek of support and/or confirmation that their concern is valid. | “Mary was not confident in her assessment of what was happening. She was kind of like, am I right? Can someone back me up here?” NM50  “It clicked with me near the end. And I was like, oh, she just wants our support in having the conversation and us supporting her and having her conversation with the manager or the doctor.” NM10 |
| Shared professional identity | Specific to nursing in this study:  Receiving from a speaker from your own discipline is easier as you know the role and know what the other person is going through. You can empathise with the speaker regarding the situation. | “It's easier to hash it out with someone if you're a similar job role, because you both understand the minute problems you face, like so you kind of both understand what delaying someone's discharge will mean to this and that.” NM76  “If it is a fellow nurse, it is like, ok, I send the team leader or whoever, it is like we collectively can get all this together.” NM14  “My whole impression that she was a caring, kind nurse, she was just like, we have all done that shift where you are just flat strapped. And there's this and this and that, and it's like hey, we're with you girl. What can we do?” NM08 |
| The importance of different perspectives | Having different disciplines present (discharge team) provides different perspectives to the same situation.  Clinical discipline impacts perception of the concern. | “We [medical officers] are thinking what the blood test shows, or that the patient is improving. We don’t often stop and think that what's going to happen with the patient behind the scenes when they go home.” MO03  “So, it just comes down to which aspect of our care is yours, everyone's slice of the pie is intrinsic to them” NM89 |
| Awareness of power differentials | Receivers are aware of potential power differentials due to profession, seniority, and/or numbers (multiple receivers and one speaker). | “In this respect, it's extra good for us just to be aware and mindful of the fact that this is someone's speaks up towards us that is much more junior, just how hard it is for them to do that.” MO08  “I'm a really junior consultant and I think I'm trying to be really mindful of that and trying to make sure that it's... because you watch some of your senior colleagues.” MO15  “[It’s] just her and a team of doctors and you put your voice forward. I'm really concerned about this patient going home. And they're like, oh no, she is prepared for discharge, I don't care what you are saying. We think she can be discharged. So, there is that whole power play, a whole team of doctors is saying that she should be going home or has planned her discharge and you are one nurse standing there going - I'm advocating for my patient.” NM63 |
| Building rapport and collegiality | For a more effective conversation, receivers deploy behaviours/moves to establish and build rapport with the speaker.  The receiver feels that the conversation is collaborative, with equal accountability between the speaker and receiver.  A view that working collaboratively flattens hierarchy. | “I think it's really important to validate someone's concerns. Because when you just say, yeah, yeah, yeah, because even if you are listening, the person doesn't think you're listening. So, for you to say I can hear that you’re concerned, is quite helpful for me, so I try and use it when I speak to other people, so to mirror. I've heard you, instead of going ok.”  MO07  “I guess because we were part of a team. I guess just making sure that, yeah, that we all had our kind of role to play to help get this patient discharged.” NM85  “When we are on the ward round, we all say who we are and that relieves the tension straight away.” NM47 |
| Listening to negate error/patient harm | Motives for why the receiver was listening to message:   - speaker has my back - need to listen to avoid potential consequences (medical error) - speaking up is important for both my and the patient’s safety. | “You don't want to be the one who sent someone home for them to come back 24 hours later.” NM17  “Because we know that from our experience that the patient needs that, you act as an advocate for the patient. And sometimes they [medical officer receiver] don't like it. But you know, something could happen [to the patient].” NM60 |
| **ENABLER - Self** | | |
| Awareness of consequences on future voice | Receivers are aware of the potential impact of their behaviour on the speaker within the immediate and/or future encounters. | “It would be scary having an audience like that coming and staring at you.” AH18  “Particularly as a new graduate, she's going to feel very vulnerable.” NM54  “We could have walked in there and said, well that's not your decision. That was a real concern, but she may not have voiced a future real concern.” AH19 |
| Listening to understand | Receiver uses active listening to seek clarification of the concern to better understand the problem, before working towards a resolution. | “If I have to make a decision, I want to know the full story.” AH19  “If you're in the right frame of mind, you can pick up on those two things [patient not fit for discharge and nurse is pressured [for bed], and dealing with both issues.” AH19  “I probably would delay and I'd be willing to take in what she's saying. I definitely wouldn't cut her off. And I'll just be like, where am I going to go with this and have that sort of thought process.” NM33 |
| Self-awareness | Receiver is aware of their own emotions or internal thoughts, and how that can potentially impact how they hear and respond to the message.    Receivers know how to, or the requirement to, manage themselves in the moment e.g., manage defensiveness. | “A lot of the times we lose focus on what's our fellow staff members and team members might be dealing with.” NM89  “I think that as the receiver there, that you have to be very focused on the internal. You have to really acknowledge the internal monologue there, because you owe it [to the other person].” MO18  “It depends on what kind of day I am having. At home before I come in, the way it is said to me.” NM82  “So, you have to be, it'd be really hard in that situation if you are tired and hungry to not attribute blame, you'd have to really think. Yeah. And put yourself in a receiving mind set and not attribute blame to the person sitting at the bedside. Probably not their fault. But I can see how you get yourself into the headspace if you weren't being careful with the way you are managing your thoughts.” MO18 |
| Knowing how to receive | Receivers describe that knowing how to respond enhances, or can enhance, the quality of the conversation. | “My instinct was to settle Mary the nurse. And then she'd do a better job.” MO11  “And this sort of program, you have better tools to manage that as well [receiving and responding].” NM54  “So, you'd say something like, I can hear where you coming from, I understand your very concerned.” NM25 |
| **ENABLER - Climate** | | |
| More than one receiver | Having a team of receivers better enables a conversation as:   - receiver responsibility is shared - speaker can receive more support with more people present. | “She [Mary] probably felt more supported than just one person because we were a team.” NM84  “With [name] jumping in and starting to ask them questions was really helpful. Because it was someone kind of taking the lead in talking back so that, you know, you didn't just kept being talked at.” NM69 |
| Presence of the patient | The conversation is enhanced by the patient being present and included. | “She (patient) wanted to be part of the conversation.” NM25  “This is a good time to include the patient.” MO02 |
| Organisational culture | The organisational culture supports speaking up. | “It becomes a bit of a culture sometimes and once that culture is broken, that whole thing changes, but sometimes it's the people up top that brings that culture in. Yes, and it's a matter that people be monitored.” NM54  “I was in intensive care and ED I think that probably the hierarchies are flattened a little, it's more collegiate.” NM25 |
